# Effects of transcutaneous electrical nerve stimulation on myocardial protection in patients undergoing aortic valve replacement: a randomized clinical trial

**DOI:** 10.1186/s12871-022-01611-x

**Published:** 2022-03-09

**Authors:** Youn Joung Cho, Dhong-Eun Jung, Karam Nam, Jinyoung Bae, Seohee Lee, Yunseok Jeon

**Affiliations:** Department of Anesthesiology and Pain Medicine, Seoul National University Hospital, Seoul National University College of Medicine, Seoul, 03080 South Korea

**Keywords:** Transcutaneous electrical nerve stimulation, Myocardial protection, Cardiac surgery, Aortic valve replacement, Ischemia reperfusion injury

## Abstract

**Background:**

Cardiopulmonary bypass-related myocardial ischemia-reperfusion injury is a major contributor to postoperative morbidity. Although transcutaneous electrical nerve stimulation (TENS) has been found to have cardioprotective effects in animal studies and healthy volunteers, its effects on cardiac surgery under cardiopulmonary bypass patients have not been evaluated. We investigated the effects of TENS on myocardial protection in patients undergoing aortic valve replacement surgery using cardiopulmonary bypass.

**Methods:**

Thirty patients were randomized to receive TENS or sham in three different anesthetic states – pre-anesthesia, sevoflurane, or propofol (each *n* = 5). TENS was applied with a pulse width of 385 μs and a frequency of 10 Hz using two surface electrodes at the upper arm for 30 min. Sham treatment was provided without stimulation. The primary outcome was the difference in myocardial infarct size following ischemia-reperfusion injury in rat hearts perfused with pre- and post-TENS plasma dialysate obtained from the patients using Langendorff perfusion system. The cardioprotective effects of TENS were determined by assessing reduction in infarct size following treatment.

**Results:**

There were no differences in myocardial infarct size between pre- and post-treatment in any group (41.4 ± 4.3% vs. 36.7 ± 5.3%, 39.8 ± 7.3% vs. 27.8 ± 12.0%, and 41.6 ± 2.2% vs. 37.8 ± 7.6%; *p* = 0.080, 0.152, and 0.353 in the pre-anesthesia, sevoflurane, and propofol groups, respectively).

**Conclusions:**

In our study, TENS did not show a cardioprotective effect in patients undergoing aortic valve replacement surgery.

**Trial registration:**

This study was registered at clinicaltrials.gov (NCT03859115, on March 1, 2019).

**Supplementary Information:**

The online version contains supplementary material available at 10.1186/s12871-022-01611-x.

## Background

Despite advancements in surgical, anesthetic, and perfusion techniques, cardiopulmonary bypass (CPB)-related myocardial ischemia-reperfusion (IR) injury is a major contributor to postoperative morbidity and mortality after cardiac surgery [1]. Current incomplete myocardial protection during CPB results in myocardial dysfunction and heart failure, particularly in high-risk patients with reduced cardiac function, and causes adverse outcomes following cardiac surgery [2].

Transcutaneous electrical nerve stimulation (TENS) is a peripheral stimulation technique that is used to apply an electrical current through the peripheral skin surface [3]. In addition to relieving pain [4], TENS improves postoperative pulmonary function and recovery [5]. Moreover, it has been found to have myocardial protective effects against IR injury in animal studies and healthy volunteers [6]. However, there is a paucity of data on the cardioprotective effects of TENS in patients undergoing cardiac surgery using CPB.

To investigate the effects of TENS on CPB-related myocardial injury particularly in aortic valve surgery patients, we hypothesized that TENS performed in patients undergoing aortic valve replacement (AVR) surgery using CPB would have myocardial protective effects. To evaluate our hypothesis, we performed TENS or a sham treatment in three different anesthetic situations, including a pre-anesthesia state, or under sevoflurane or propofol anesthesia in patients undergoing elective AVR surgery. To determine the cardioprotective effects of TENS, we compared the myocardial infarct (MI) size of isolated rat hearts perfused with plasma dialysate from patients receiving TENS or the sham treatment, using the Langendorff heart IR injury model.

## Methods

### Ethics

This study was approved by the Institutional Review Board of Seoul National University Hospital (#1901–158-1006) and was registered at clinicaltrials.gov (NCT03859115, on March 1, 2019) before patient enrollment. The study was performed following Good Clinical Practice guidelines and the principles of the Declaration of Helsinki and adhered to Consolidated Standards of Reporting Trials (CONSORT) guidelines. All participants provided written informed consent and were allowed to reverse their consent at any time. All animal experiments were approved by the Institutional Animal Care and Use Committee (IACUC) of Seoul National University (#SNU-190306-2) and were performed following the ARRIVE guidelines and the Guide for the Care and Use of Laboratory Animals by the US National Institutes of Health (NIH, Bethesda, MD, USA).

### Study participants and randomization

**Fig. 1:**
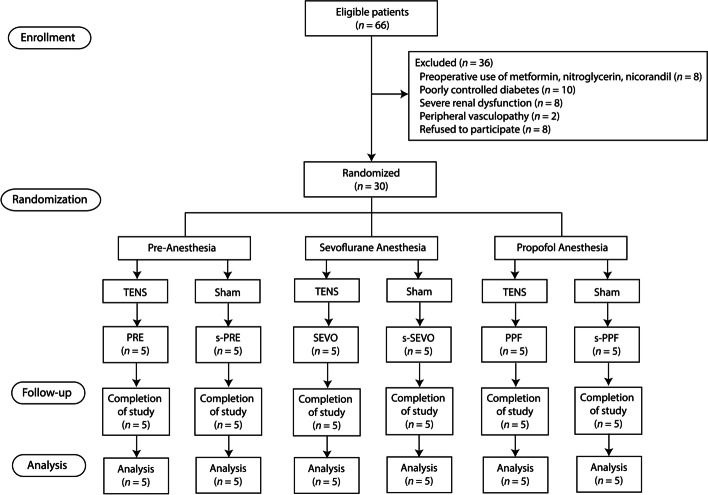
CONSORT flow diagram. TENS, transcutaneous electrical nerve stimulation; PRE, pre-anesthesia; s-, sham; SEVO, sevoflurane; PPF, propofol

Eligible patients were adult patients (age ≥ 20 years) scheduled for AVR surgery using CPB. Patients who used metformin, nitroglycerin, or nicorandil preoperatively, which could interfere with the effects of preconditioning or TENS; loss of intact skin or severe discomfort with the TENS procedure; poorly controlled hypertension or diabetes; severely impaired renal or hepatic function; peripheral vasculopathy or neuropathy; refusal to participate; or pregnancy were excluded from the study (Fig. 1). The enrolled patients were randomized to one of the following six groups: TENS in the pre-anesthesia state (PRE), sham in the pre-anesthesia state (s-PRE), TENS under sevoflurane anesthesia (SEVO), sham under sevoflurane anesthesia (s-SEVO), TENS under propofol anesthesia (PPF), and sham under propofol anesthesia (s-PPF). Block randomization (blocks of six) was performed using a computer-generated randomization program by an independent researcher to allocate patients in a 1:1:1:1:1:1 ratio. Group assignments were concealed in opaque envelopes and blinded to the surgeons and investigators performing the animal experiments and data analyses. All animal studies and data analyses were undertaken by investigators blinded to the treatment allocation.

### Study protocol

Patients were monitored via five-lead electrocardiogram, noninvasive blood pressure, pulse oximetry, bispectral index, and regional cerebral oximetry using near-infrared spectroscopy. The radial artery was cannulated to monitor arterial blood pressure continuously.

**Fig. 2:**
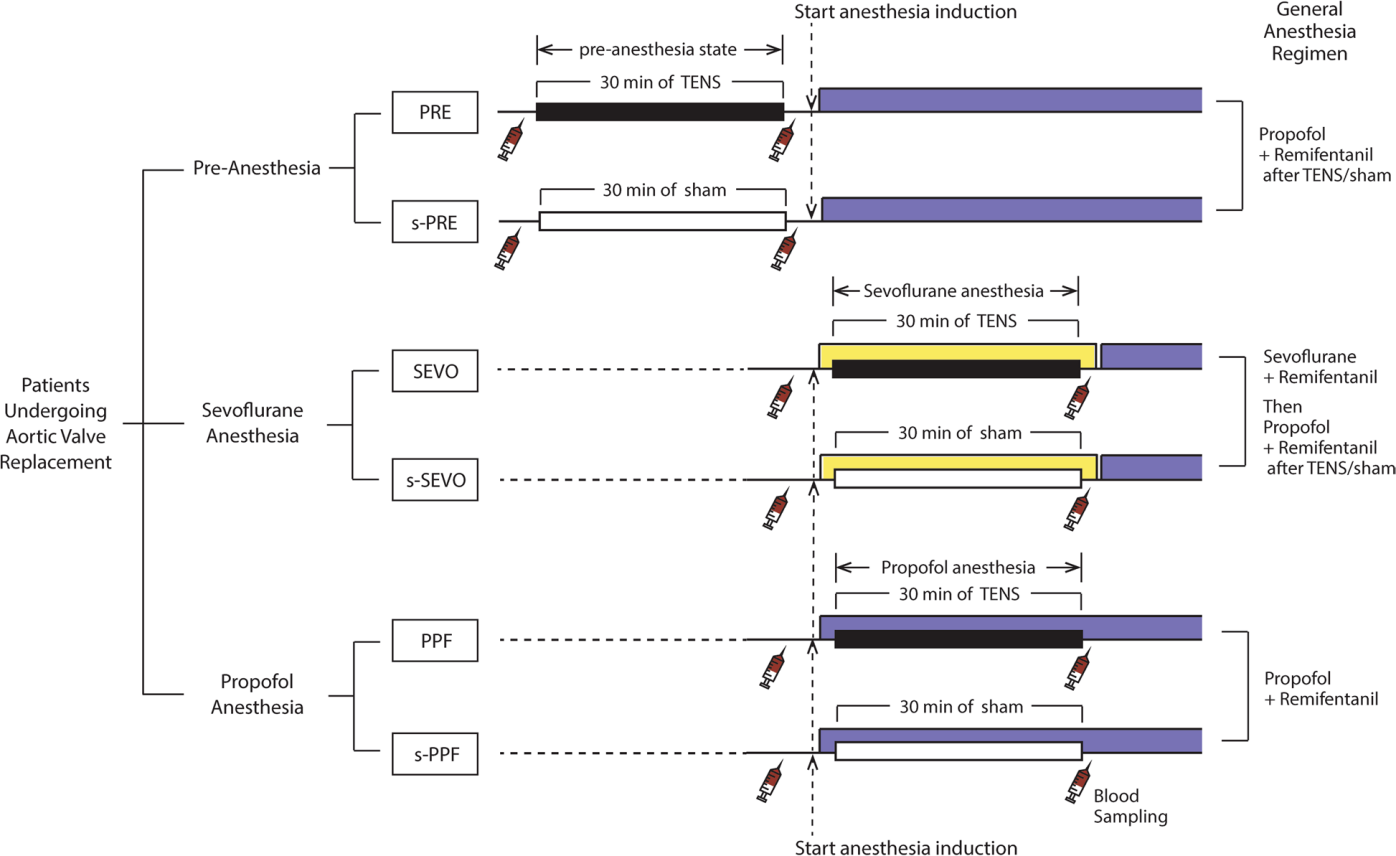
Study flow. PRE, pre-anesthesia; s-, sham; SEVO, sevoflurane; PPF, propofol; TENS, transcutaneous electrical nerve stimulation

In the pre-anesthesia groups (PRE and s-PRE), TENS or sham treatment was performed in the awake state before inducing anesthesia. Two surface electrodes (5 × 5 cm) were attached to the skin of the anterior surface of the upper arm coinciding with the C5 dermatome [6]. A dual-channel TENS device (model CLFS-200, CAS Inc., Yangju-si, Gyeonggi-do, South Korea) was set to the beating mode with a pulse width of 385 μs and a frequency of 10 Hz. The maximum output of the device was 2 mA. The maximal tolerable intensity of the stimulus was adjusted for each patient before treatment to the maximum level that the subject felt a strong but comfortable tingling sensation. The stimulus was applied for 30 min in the TENS groups, while the device was not turned on for 30 min in the sham groups (Fig. 2).

In the sevoflurane groups (SEVO and s-SEVO), general anesthesia was induced by administration of midazolam (0.1 mg/kg) and sufentanil (1 μg/kg) and was maintained by inhalation of 1.5–2 vol% sevoflurane (Sojourn, Piramal Critical Care Inc., Bethlehem, PA, USA). Target-controlled infusion of remifentanil (effect-site concentration of 1–6 ng/mL) was used for adequate control of the hemodynamic response. The TENS or the sham treatment was provided under sevoflurane anesthesia in the sevoflurane groups. In the propofol groups (PPF and s-PPF), anesthesia was induced and maintained with target-controlled infusion of propofol (Fresofol 2 MCT 2%, Fresenius Kabi, Graz, Austria; effect-site concentration of 2–4 μg/mL) and remifentanil. The TENS or sham treatment was provided under propofol anesthesia in the propofol groups. After completing the TENS/sham treatment, anesthesia was maintained with propofol and remifentanil in all groups (Fig. 2). Perioperative variables, including transfusion and postoperative in-hospital outcome data (e.g., atrial fibrillation, re-exploration, stroke, myocardial infarction, and mortality) were assessed.

### Aortic valve replacement and cardiopulmonary bypass strategy

AVR was performed using aortic and bicaval cannulation via a median sternotomy. Prosthetic valves were implanted after removing the native valve leaflets and decalcifying the annulus. The suture technique was identical in almost all patients: non-everting mattress sutures buttress-reinforced with polytetrafluoroethylene as a tubule or a pledget were used.

The CPB circuit incorporating a membrane oxygenator was primed with Ringer’s lactate solution, mannitol, 20% albumin, sodium bicarbonate, and heparin. Following systemic heparinization, non-pulsatile bypass was performed with a pump flow of 2–2.5 L/min/m^2^ body surface area; α-stat pH management was implemented under mild hypothermia (28–32 °C) or moderate hypothermia (23–28 °C) when aorta surgery was combined. Activated clotting time was maintained at > 400 s and intraoperative cell salvage was used during bypass. Myocardial protection was supported by antegrade or retrograde cold cardioplegic arrest. Custodiol® histidine-tryptophan-ketoglutarate (HTK) solution (Essential Pharmaceuticals, LLC, Ewing, NJ, USA) or Del Nido cardioplegia was used following surgeons’ preference. The HTK solution was infused as a single dose of 20 mL/kg, and Del Nido cardioplegia was used as an initial dose of 1000 mL following additional dose of 500 mL when the aortic cross clamp time exceeded 90 min. Heparinization was reversed with protamine after weaning from CPB.

### Preparation of plasma dialysate

**Fig. 3:**
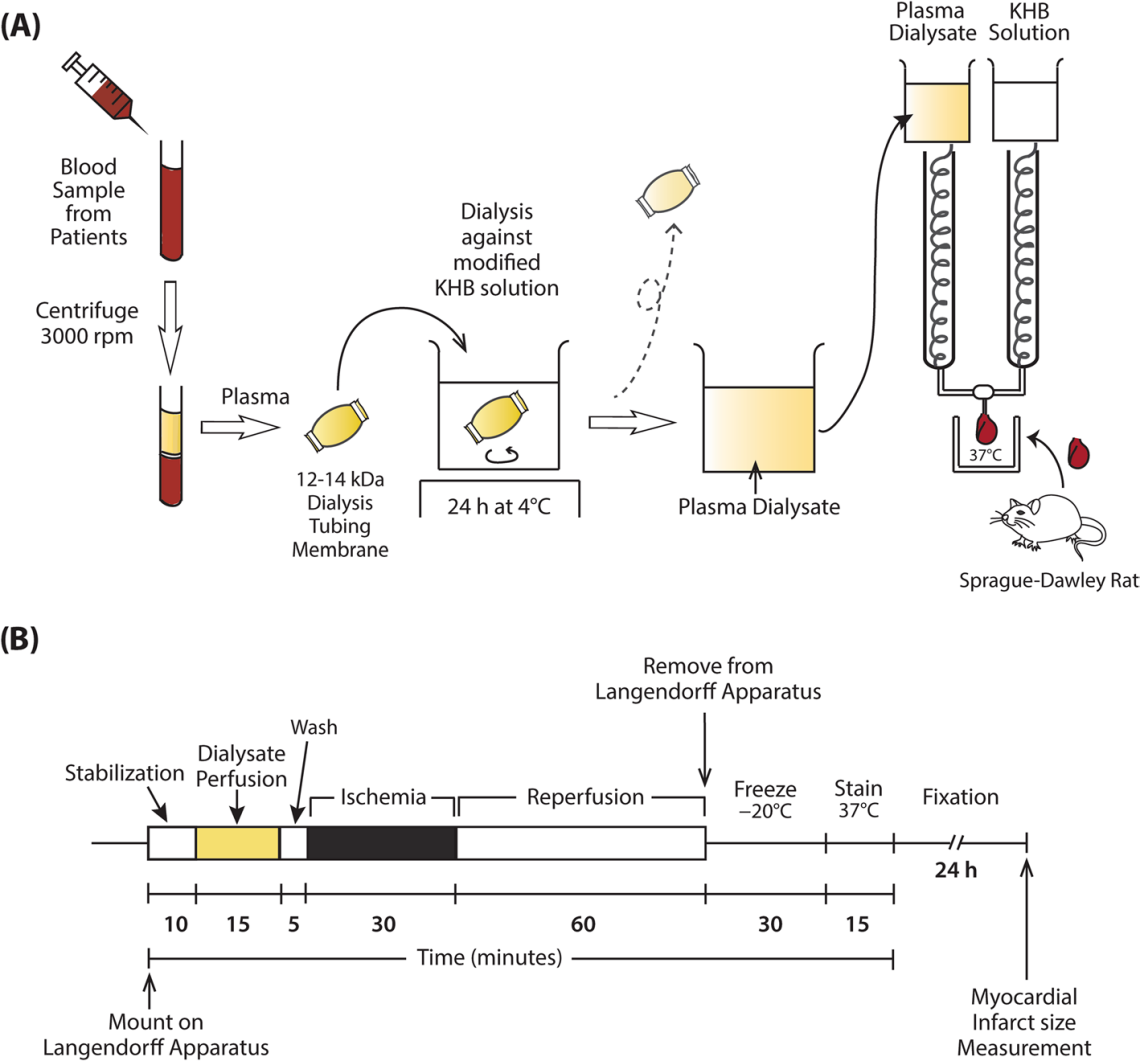
**A** Langendorff apparatus for rat heart ischemia-reperfusion injury model. **B** Ischemia-reperfusion injury protocol using Langendorff rat heart model. KHB, Krebs-Henseleit buffer

In all, 30 mL samples of whole blood were obtained before and after each TENS/sham treatment to prepare the plasma dialysate (Fig. 2). The blood was obtained after TENS/sham treatment to investigate the pure effects of each treatment and minimize confounding factors related to surgical procedures or inflammatory response to the use of CPB. The blood was collected in sodium heparin tubes and centrifuged at 3000 rpm at room temperature for 20 min to obtain the plasma. The plasma fraction was carefully obtained without disturbing the buffy coat and was contained in the dialyzing tubing with a 12–14 kDa cutoff membrane (Spectra/Por, Spectrum Laboratories, Inc., Rancho Dominguez, CA, USA). The plasma was dialyzed against a 20-fold volume of modified Krebs-Henseleit buffer (KHB) solution while stirring for 24 h at 4 °C (Fig. 3A). The modified KHB solution consisted of 118 mM NaCl, 4.7 mM KCl, 1.1 mM MgSO_4_·7H_2_O, 1.2 mM KH_2_PO_4_, and 1.8 mM CaCl_2_·2H_2_O. If the plasma could not be used immediately, it was stored at − 80 °C for later use. Before perfusing the rat heart, the dialysate was supplemented with 25 mM NaHCO_3_ and 11 mM D-glucose, and filtered through a 0.2 μm filter. The dialysate was equilibrated with a 95% O_2_–5% CO_2_ mixture and adjusted to a pH of 7.35–7.45 at 37 °C.

### Langendorff rat heart ischemia-reperfusion injury model

Male Sprague-Dawley rats (aged 9–11 weeks and weighing 250–350 g) were used for the Langendorff rat heart IR injury model. All animals were commercially obtained (KOATECH Corp.; Pyeongtaek-si, Gyeonggi-do, South Korea) and cared for in compliance with the Guidelines for the Care and Use of Laboratory Animals issued by the IACUC of Seoul National University. The rats were housed under specific-pathogen-free conditions on a 12-h/12-h light/dark cycle with free access to food and water. The temperature and humidity were maintained at 24–25 °C and 40–60%, respectively.

The rats were anesthetized with 6–8 vol% of inhaled sevoflurane, and their hearts were quickly excised via a clamshell thoracotomy and mounted on the Langendorff apparatus with cannulation of ascending aorta. Heparin was injected before excising the heart. The heart was perfused with KHB solution in a retrograde, non-recirculating manner through the aortic cannulation. The KHB consisted of 118 mM NaCl, 4.7 mM KCl, 25 mM NaHCO_3_, 11 mM D-glucose, 1.1 mM MgSO_4_·7H_2_O, 1.2 mM KH_2_PO_4_, and 1.8 mM CaCl_2_·2H_2_O. All perfusates were gassed with a 95% O_2_–5% CO_2_ mixture and adjusted to a pH of 7.35–7.45 at 37 °C. After 10-min stabilization, the hearts were perfused with pre- or post-treatment dialysate for 15 min, washed with KHB for 5 min, and subjected to 30 min of no-flow global ischemia and subsequent 60 min of reperfusion (Fig. 3B). The temperature of the heart was maintained at 37 °C throughout the protocol. After reperfusion, the hearts were removed from the apparatus, placed at − 20 °C for 30 min, and then cut into 5–6 slices transversely, each 1–2 mm thick using a rat heart slicer matrix. The slices were stained with 1% 2,3,5-triphenyltetrazolium chloride in 0.1 M sodium phosphate buffer, adjusted to pH 7.4 at 37 °C for 15 min. The stained slices were fixed in 10% formalin for 24 h to enhance contrast (Fig. 3B). Then, both sides of the slices were digitally scanned for planimetric analyses using ImageJ software (version 1.51, NIH) by a blinded investigator. The viable tissue was identified as brick red, and the infarcted tissue was pale white. As the hearts were subjected to global ischemia, the total cross-sectional left ventricle (LV) areas were defined as the total areas at risk, and MI size was expressed as a percentage of the total area of the LV. Each dialysate was used to perfuse only one rat heart, and the reported MI size was the mean of all measurements of all individual slices from each heart. Animal experiments were excluded from analyses if they met one of the following exclusion criteria: time to perfusion > 3 min; unstable ventricular contraction or significant ventricular arrhythmia > 3 min; or heart rate < 100 or > 400 beats per minute. The rats were euthanized by extracting the hearts and cessation of the circulation in anesthetized state during the initial step of the experimental protocol.

### Study endpoint and sample size calculation

The primary endpoint of the study was the difference in MI size after IR injury of the rat heart perfused using post-TENS dialysate compared to pre-TENS dialysate. We calculated sample size based on a previous study, in which the baseline MI size using plasma dialysate from cardiac surgical patients was 38.6 ± 3.6% [7]. If we assumed that a 30% reduction in MI size between pre- and post-TENS treatment was experimentally significant, four patients per group were required at an alpha error of 5% and a power of 95% compared using a paired t-test and G*power (version 3.1.9.2, Franz Faul, Universitat Kiel, Germany). Considering a 20% dropout rate, we calculated that five patients would be required per group, which would be a total of 30 patients in six groups for the study.

In each group, ten rats were required: five rats to determine the baseline MI size before the treatment (perfused with pre-TENS/sham dialysate), and five rats to evaluate the effects of the treatment (perfused with post-TENS/sham dialysate).

### Statistical analyses

The normality of the data was tested using the Kolmogorov-Smirnov and Shapiro-Wilk tests. According to the data distribution, continuous variables were expressed as mean ± SD or median (interquartile range) and were compared using the independent t-test, Mann–Whitney U–test, or Kruskal-Wallis test. Pre- and post-treatment values in each group were compared using the paired t-test or the Wilcoxon signed-rank test. Categorical variables were expressed as numbers (proportions) and were compared using Pearson’s chi-square or Fisher’s exact tests.

All analyses were performed using SPSS (version 21.0, IBM Corp., Armonk, NY, USA) and R software (version 3.4.3, R Development Core Team, Vienna, Austria) for Microsoft Windows. A *p* value < 0.05 was considered significant.

## Results

### Study population

Patients were enrolled between March 15, 2019 and April 8, 2020. Among 66 eligible patients, 36 were excluded due to preoperative use of metformin, nitroglycerin, or nicorandil (*n* = 8); poorly controlled diabetes (*n* = 10); severe renal dysfunction (*n* = 8); peripheral vasculopathy (*n* = 2); and refusal to participate (*n* = 8). Thirty patients were randomized into the six groups and completed the study protocol (Fig. 1).

The baseline patient characteristics are presented in Table 1 and Supplementary Table 1. Perioperative variables are presented in Table 2. The mean age of the patients was 66 ± 10 years, including 57% (17/30) male participants. There was a difference in duration of CPB among groups, but durations of aorta cross-clamp were similar (Table 2). Preoperative and postoperative levels of cardiac troponin I were not different among the groups (Tables 1 and 2).

**Table 1:** Baseline characteristics of patients undergoing aortic valve replacement

Characteristics	PRE (***n*** = 5)	s-PRE (***n*** = 5)	SEVO (***n*** = 5)	s-SEVO (***n*** = 5)	PPF (***n*** = 5)	s-PPF (***n*** = 5)	***p***-value
Age (yr)	66 ± 15	70 ± 8	65 ± 5	62 ± 11	68 ± 10	64 ± 14	0.850
	61 (57–77	73 (63–76	65 (64–66	59 (53–73	69 (68–71	67 (63–71	0.966
	[50–85])	[60–78])	[57–71])	[50–75])	[53–81])	[42–79])	
Male sex	2 (40%)	3 (60%)	1 (20%)	3 (60%)	2 (40%)	2 (40%)	0.950
Height (cm)	159 ± 8	157 ± 8	161 ± 9	162 ± 13	159 ± 13	157 ± 14	0.658
Weight (kg)	65.7 ± 12.5	55.3 ± 9.0	60.8 ± 13.2	66.8 ± 16.5	57.4 ± 10.3	56.3 ± 11.5	0.139
BMI (kg/m^2^)	25.8 ± 2.7	22.3 ± 2.4	23.3 ± 3.6	25.1 ± 3.2	22.6 ± 2.0	22.5 ± 2.0	0.786
BSA (m^2^)	1.7 ± 0.2	1.6 ± 0.2	1.6 ± 0.2	1.7 ± 0.3	1.6 ± 0.2	1.6 ± 0.2	0.428
Smoker	2 (40%)	1 (20%)	0 (0%)	1 (20%)	2 (40%)	2 (40%)	0.808
Baseline LV EF (%)	64 ± 5	59 ± 6	61 ± 10	59 ± 7	60 ± 7	65 ± 6	0.950
Baseline troponin I (ng/ml)	0.01 (0.01–0.03)	0.01 (0.01–0.03)	0.01 (0.01–0.11)	0.01 (0.01–0.05)	0.01 (0.01–0.06)	0.01 (0.01–0.02)	0.950
Baseline hematocrit (%)	39 ± 4	39 ± 7	36 ± 7	41 ± 6	39 ± 7	38 ± 3	0.938
Baseline eGFR (mL/min/1.73 m^2^)	78.9 ± 17.4	75.9 ± 33.8	77.9 ± 14.0	82.5 ± 22.9	87.6 ± 15.5	79.2 ± 13.2	0.850
Operation							0.956
AVR only	2 (40%)	2 (40%)	3 (60%)	1 (20%)	1 (20%)	3 (60%)	
AVR + ascending aorta surgery	2 (40%)	2 (40%)	1 (20%)	2 (40%)	2 (40%)	1 (20%)	
AVR + mitral valve surgery or CABG	1 (20%)	1 (20%)	1 (20%)	2 (40%)	2 (40%)	1 (20%)	

Data are presented as mean ± SD, median (interquartile range [range]), or number (%)

*TENS* transcutaneous electrical nerve stimulation, *SEVO* sevoflurane, *PPF* propofol, *BMI* body mass index, *BSA* body surface area, *LV EF* left ventricular ejection fraction, *eGFR* estimated glomerular filtration rate, *AVR* aortic valve replacement, *CABG* coronary artery bypass graft

**Table 2:** Perioperative variables in patients undergoing aortic valve replacement surgery

	PRE (***n*** = 5)	s-PRE (***n*** = 5)	SEVO (***n*** = 5)	s-SEVO (***n*** = 5)	PPF (***n*** = 5)	s-PPF (***n*** = 5)	***p***-value
Operation duration (min)	314 ± 49	326 ± 82	219 ± 54	321 ± 82	227 ± 43	288 ± 108	0.089
Anesthesia duration (min)	370 ± 52	400 ± 87	279 ± 50	380 ± 85	290 ± 42	347 ± 109	0.102
Cardiopulmonary bypass duration (min)	171 ± 38	177 ± 61	111 ± 37	163 ± 51	107 ± 11	125 ± 33	0.036
Aorta cross-clamp duration (min)	130 ± 44	111 ± 49	85 ± 30	109 ± 52	72 ± 7	83 ± 29	0.193
Type of cardioplegia							0.004
HTK solution	5 (100%)	3 (60%)	1 (20%)	1 (20%)	0 (0%)	0 (0%)	
Del Nido	0 (0%)	2 (40%)	4 (80%)	4 (80%)	5 (100%)	5 (100%)	
Total dosage of cardioplegia (L)	4.0 (4.0–4.5)	3.0 (1.5–4.5)	2.0 (1.1–3.0)	1.3 (1.2–4.4)	1.2 (1.0–1.8)	1.2 (1.0–1.8)	0.037
Nadir nasal temperature (°C)	27.6 ± 1.7	29.2 ± 1.8	29.4 ± 2.0	29.6 ± 2.9	30.6 ± 1.8	29.3 ± 2.6	0.388
Nadir rectal temperature (°C)	29.1 ± 2.0	29.9 ± 1.5	30.8 ± 0.6	30.1 ± 3.0	31.8 ± 0.8	30.5 ± 2.4	0.266
Intraoperative RBC transfusion (unit)	0 (0–0 [0–0])	0 (0–0 [0–0])	0 (0–1 [0–1])	0 (0–1 [0–1])	0 (0–0 [0–1])	0 (0–0 [0–2])	0.477
Intraoperative FFP transfusion (unit)	0 (0–2 [0–3])	3 (2–3 [0–3])	2 (0–3 [0–3])	0 (0–3 [0–3])	0 (0–0 [0–0])	0 (0–3 [0–3])	0.271
Intraoperative plateletpheresis transfusion (unit)	0 (0–0 [0–1])	1 (0–1 [0–1])	0 (0–1 [0–2])	0 (0–1 [0–2])	0 (0–0 [0–0])	0 (0–0 [0–1])	0.425
Postoperative RBC transfusion (unit)	2 (1–2 [0–5])	2 (1–3 [0–8])	0 (0–0 [0–5])	2 (0–2 [0–3])	0 (0–1 [0–2])	1 (0–3 [0–4])	0.507
Postoperative FFP transfusion (unit)	0 (0–2 [0–4])	1 (0–2 [0–3])	0 (0–0 [0–4])	0 (0–0 [0–1])	0 (0–0 [0–1])	0 (0–1 [0–1])	0.626
Postoperative plateletpheresis transfusion (unit)	1 (0–1 [0–2])	2 (1–2 [0–6])	0 (0–0 [0–1])	0 (0–0 [0–3])	0 (0–0 [0–1])	0 (0–1 [0–2])	0.194
Peak postoperative troponin I within 72 h (ng/mL)	9.31 (7.38–38.51)	24.92 (6.95–185.85)	4.31 (2.61–19.93)	13.35 (4.21–92.68)	6.69 (3.48–7.93)	6.18 (4.61–27.01)	0.317
Postoperative atrial fibrillation	0 (0%)	1 (20%)	1 (20%)	1 (20%)	2 (40%)	1 (20%)	0.974
Re-exploration	0 (0%)	0 (0%)	0 (0%)	0 (0%)	0 (0%)	0 (0%)	N/A
In-hospital stroke	0 (0%)	0 (0%)	1 (20%)	1 (20%)	0 (0%)	0 (0%)	0.509
In-hospital myocardial infarction	0 (0%)	0 (0%)	0 (0%)	0 (0%)	0 (0%)	0 (0%)	N/A
In-hospital mortality	0 (0%)	0 (0%)	0 (0%)	0 (0%)	0 (0%)	0 (0%)	N/A

Data are presented as mean ± SD, median (interquartile range [range]), or number (%)

*TENS* transcutaneous electrical nerve stimulation, *SEVO* sevoflurane, *PPF* propofol, *HTK* histidine-tryptophan-ketoglutarate, *RBC* red blood cell, *FFP* fresh frozen plasma

No complications or morbidities related to TENS or the sham treatment were observed in any patient. A total of 60 rats were used for the animal experiments, and no experiment was excluded according to the pre-defined criteria.

### Study outcomes

**Fig. 4:**
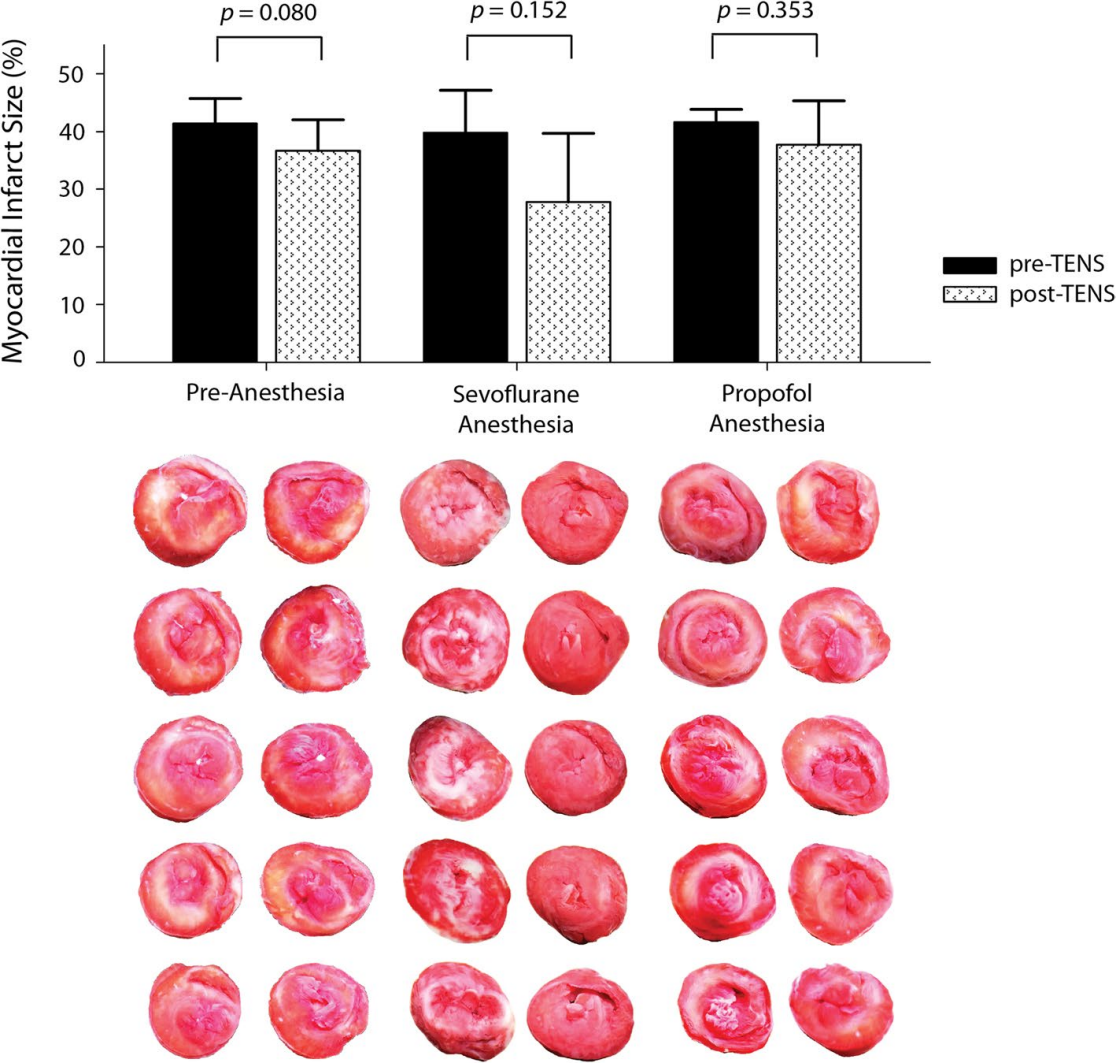
Comparisons of rat myocardial infarct size before and after TENS in three different anesthesia groups. TENS, transcutaneous electrical nerve stimulation

**Fig. 5:**
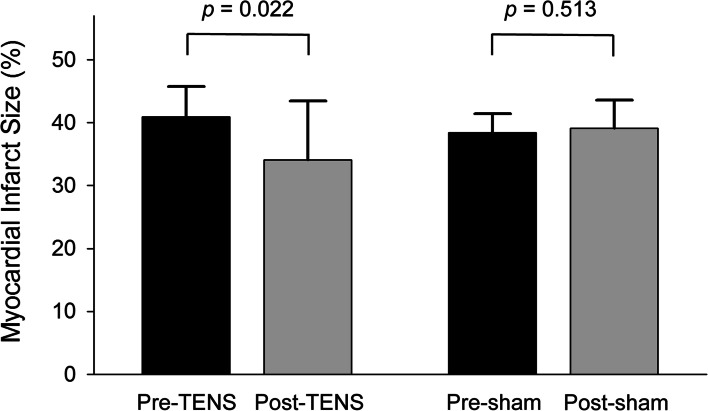
Myocardial infarct size of rat hearts following pre- and post-TENS/sham treatment regardless of anesthetic state. TENS, transcutaneous electrical nerve stimulation

No differences in MI size were observed between pre- and post-sham treatment in the three anesthesia groups (41.2 ± 2.6% vs. 41.2 ± 4.9%, 36.8 ± 1.3% vs. 37.1 ± 5.1%, and 37.3 ± 3.0% vs. 39.0 ± 3.2% in the s-PRE, s-SEVO, and s-PPF groups; *p* = 0.982, 0.925, and 0.152, respectively). The primary endpoint, the difference in MI size between pre- and post-TENS treatment, was not significantly different in any of the three groups (41.4 ± 4.3% vs. 36.7 ± 5.3%, 39.8 ± 7.3% vs. 27.8 ± 12.0%, and 41.6 ± 2.2% vs. 37.8 ± 7.6% in the PRE, SEVO, and PPF groups; *p* = 0.080, 0.152, and 0.353, respectively; Fig. 4). For all TENS groups (*n* = 15), post-TENS MI size was significantly smaller compared to pre-TENS MI size (34.1 ± 9.3% vs. 40.9 ± 4.8%, *p* = 0.022; Fig. 5). In all sham groups (*n* = 15), pre- and post-sham MI sizes were not different (38.4 ± 3.0% vs. 39.1 ± 4.5%, *p* = 0.513; Fig. 5).

No significant differences were observed in transfusion and in-hospital outcomes in any of the groups (Table 2).

## Discussion

We did not observe myocardial protective effects of TENS in patients undergoing AVR surgery. No differences in MI size of perfused rat hearts were observed with the pre- and post-treatment dialysate in patients who received TENS or the sham in any of the three anesthetic states of pre-anesthesia, sevoflurane, or propofol.

Myocardial IR injury commonly occurs following cardiac surgery and CPB [1]. Aortic cross-clamping, cardioplegic arrest, and the subsequent systemic inflammatory response contribute to myocardial IR injury and organ dysfunction [8]. Numerous myocardial protective strategies have been developed and adopted for cardiac surgery [2]. However, the inevitable process of myocardial IR injury following CPB and cardiac surgery, and the consequent myocardial dysfunction result in serious postoperative morbidity and mortality [1, 8]. Therefore, clinicians and researchers have devoted extensive efforts to reduce myocardial IR injury and subsequent dysfunction in patients undergoing CPB and cardiac surgery.

Preconditioning is a beneficial organ-protective effect against IR injury [9, 10]. Repeated sublethal ischemic insults on remote organs before subsequent lethal ischemia of the target tissue, which is referred to as remote ischemic preconditioning (RIPC), has been found to reduce myocardial damage in animal experiments [11] and clinical studies [12]. The protective mechanism of RIPC is deemed to be related to both humoral and neural factors [13]. The neural pathways associated with RIPC involve sensory afferent nerves and vagal efferent nerves [14]. Peripheral sensory neuronal pathways are activated when a remote stimulus is provided, with an associated release of humoral factors and activation of the efferent nerves [15].

As RIPC attenuates myocardial injury, TENS or electrical acupoint stimulation shows comparable cardioprotection [6]. Electroacupuncture applied to the forelimbs of rabbits attenuates myocardial damage via perfusion with dialysate, and the cardioprotective effects of electroacupuncture were similar to that of RIPC [16]. Transcutaneous electric acupoint stimulation of the forearm also attenuates myocardial injury by reducing cardiac troponin I in pediatric patients undergoing cardiac surgery [17]. Moreover, the cardioprotective effects mediated by dialysate from patients who received RIPC is abolished in the presence of diabetic neuropathy [18]. Therefore, the neural pathway plays a key role in the cardioprotective mechanism against IR injury through the afferent somatic nervous stimulation and efferent nerves innervating the heart and other target organs [19].

TENS uses patient-tolerable electric stimulation of peripheral nerves to induce pain relief, inhibit the inflammatory response, and enhance recovery of organ function [5, 20–22]. TENS has been categorized into conventional (low-intensity and high-frequency), acupuncture-like (high-intensity and low-frequency), and intense TENS types (high-intensity and high-frequency) [23]. The protocol used in the present study is similar to acupuncture-like TENS, which is characterized by low-frequency (2–4 Hz), high intensity (to tolerance threshold), and longer pulse width (100–400 μs). Acupuncture-like TENS stimulates small diameter, high threshold peripheral afferent (A-delta) neurons and activates the extrasegmental descending pain inhibitory pathway [23].

However, there is a discrepancy in the results of cardioprotective therapies between animal experiments and clinical studies involving heterogenous diseased patients [24]. Among the suggested confounders, the anesthetic regimen used during RIPC interferes with the cardioprotective effects induced by RIPC [25]. The effects of RIPC are inconsistent according to the anesthetic agents administered during the procedure [26, 27]. RIPC reduces MI size of rat hearts when perfused with dialysate from patients who received RIPC in the pre-anesthetic state, while RIPC has no effect under propofol or sevoflurane anesthesia [7]. Therefore, we evaluated the effects of TENS in three different anesthesia states, including pre-anesthesia, sevoflurane, or propofol anesthesia in this study.

We observed no effects of TENS on myocardial protection in patients without anesthesia, or under either sevoflurane or propofol anesthesia. The post-TENS MI size was smaller than the pre-TENS MI size in all patients who received TENS, while no difference was observed between pre-sham and post-sham MI sizes, regardless of anesthesia state. Cardiac biomarker (troponin I) changes were well correlated with the results of experimental myocardial damage and its extent, showing no difference among the groups. There are several explanations for the lack of a TENS cardioprotective effect in this study. First, the electrical stimulation protocol varies among studies [6, 16, 17, 28, 29]. An extended duration of treatment such as 5 consecutive days using multiple simulating points elicits electoacupuncture cardioprotective effects in patients undergoing heart valve surgery [28]. The stimulation can be provided for 30 or 60 min according to the study protocol [16, 17]. We used a similar stimulation intensity as several previous studies [16, 28, 30, 31], while other investigators used more intense stimulation in different study subjects (healthy volunteers vs. cardiac valve disease patients) [6]. As the mechanism of the cardioprotective effects of TENS or a unified protocol has not been established, differences in the TENS protocol may affect treatment efficacy in different situations.

Second, the electrical stimulating point has varied among studies. We used the same stimulating point (C5 dermatome) as used in a previous study, in which TENS exhibited effective myocardial protection in animal hearts and healthy volunteers [6]. This area also partly overlapped with a previously described point (Neiguan or PC6) in other studies, which is located at the anterior forearm, and the electrical stimulation reduced myocardial IR injury [16, 17, 28, 31, 32]. Other effective stimulation points involve the Lieque (LU7) or Yunmen (LU2) point, which is also located on the upper extremities [28]. Electrical stimulation of the femoral nerve reduces MI size following myocardial IR injury in urethane-anesthetized rats [33]. The auricular branch of the vagus nerve or tragus stimulation also produces cardioprotection in canine MI models [21, 29]. Whereas, the location of the stimulation did not affect pain intensity in healthy volunteers, suggesting nonspecific effects of TENS [34]. Therefore, whether the location of electrical stimulation affects TENS results remains to be investigated.

Third, the study population in this study consisted of aged patients with concomitant underlying diseases and multiple medications. The hearts of old rats cannot be preconditioned and those of middle-aged rats have blunted responses compared to those of young adults [35]. Therefore, there may be defects in the signaling cascades within aged hearts. Transcutaneous electrical acupoint stimulation effectively reduces postoperative troponin levels in pediatric patients undergoing open-heart surgery [17]. The population in that study had a mean age of 4 years and had fewer comorbidities and medications than the aged patients with degenerative valve diseases in our study. Upper limb TENS in healthy relatively younger (25–32 years old) non-smoking volunteers also results in a greater reduction in MI size of isolated animal hearts using dialysate [6]. Moreover, the use of beta-blocking agents diminishes the effects of electric acupuncture on reducing the MI size of rat hearts [36]. Therefore, aging, comorbidities and medications, which may affect signaling cascades through neural pathways, might have hindered the effects of TENS in our study. Additionally, although CPB durations were different among groups, the durations of aorta cross-clamp, which may reflect ischemic period, were similar and may have little effect on TENS treatment in this study.

This study had some limitations. Although we calculated the sample size based on a previous study, we evaluated the effects of TENS in a limited number of patients. Moreover, the MI size of the rat hearts decreased after TENS when we put all TENS patients together regardless of anesthetic state, while there were no differences in the sham patients. Therefore, more patients may have been required to control the confounding variables. Moreover, considering the effects of TENS on rat myocardium, these treatment effects may worth be evaluated in the coronary artery disease group in future studies.

Although we did not observe any treatment effects even in the pre-anesthesia state, we performed this study using a well-defined protocol, and our results provide evidence and information for further investigations on this topic.

## Conclusions

In conclusion, TENS did not show a cardioprotective effect in patients undergoing AVR surgery.

## Supplementary Information

**Additional file 1: Supplementary Table 1**. Comorbidities, preoperative medications and aortic valve characteristics of the included patients.

## Data Availability

The data set used and analyzed during the current study are available from the corresponding author on reasonable request.

## Abbreviations

CPB: Cardiopulmonary bypass
IR: Ischemia-reperfusion
TENS: Transcutaneous electrical nerve stimulation
AVR: Aortic valve replacement
MI: Myocardial infarct
CONSORT: Consolidated Standards of Reporting Trials
IACUC: Institutional Animal Care and Use Committee
PRE: TENS in the pre-anesthesia state
s-PRE: sham in the pre-anesthesia state
SEVO: TENS under sevoflurane anesthesia
s-SEVO: sham under sevoflurane anesthesia
PPF: TENS under propofol anesthesia
s-PPF: sham under propofol anesthesia
HTK: Histidine-tryptophan-ketoglutarate
KHB: Krebs-Henseleit buffer
LV: Left ventricle
RIPC: Remote ischemic preconditioning

## References

[1] Royster RL (1993). Myocardial dysfunction following cardiopulmonary bypass: recovery patterns, predictors of inotropic need, theoretical concepts of inotropic administration. J Cardiothorac Vasc Anesth.

[2] De Hert S, Moerman A (2015). Myocardial injury and protection related to cardiopulmonary bypass. Best Pract Res Clin Anaesthesiol.

[3] Sluka KA, Walsh D (2003). Transcutaneous electrical nerve stimulation: basic science mechanisms and clinical effectiveness. J Pain.

[4] Bayindir O, Paker T, Akpinar B, Erenturk S, Askin D, Aytac A (1991). Use of transcutaneous electrical nerve stimulation in the control of postoperative chest pain after cardiac surgery. J Cardiothorac Vasc Anesth.

[5] Cipriano G, de Camargo Carvalho AC, Bernardelli GF, Tayar Peres PA (2008). Short-term transcutaneous electrical nerve stimulation after cardiac surgery: effect on pain, pulmonary function and electrical muscle activity. Interact Cardiovasc Thorac Surg.

[6] Merlocco AC, Redington KL, Disenhouse T (2014). Transcutaneous electrical nerve stimulation as a novel method of remote preconditioning: in vitro validation in an animal model and first human observations. Basic Res Cardiol.

[7] Cho YJ, Nam K, Kim TK (2019). Sevoflurane, Propofol and Carvedilol block myocardial protection by limb remote ischemic preconditioning. Int J Mol Sci.

[8] Murphy GJ, Angelini GD (2004). Side effects of cardiopulmonary bypass: what is the reality?. J Card Surg.

[9] Heusch G, Botker HE, Przyklenk K, Redington A, Yellon D (2015). Remote ischemic conditioning. J Am Coll Cardiol.

[10] Zhou H, Yang L, Wang G (2019). Remote ischemic preconditioning prevents postoperative acute kidney injury after open total aortic arch replacement: a double-blind, randomized, sham-controlled trial. Anesth Analg.

[11] Przyklenk K, Bauer B, Ovize M, Kloner RA, Whittaker P (1993). Regional ischemic 'preconditioning' protects remote virgin myocardium from subsequent sustained coronary occlusion. Circulation..

[12] Botker HE, Kharbanda R, Schmidt MR (2010). Remote ischaemic conditioning before hospital admission, as a complement to angioplasty, and effect on myocardial salvage in patients with acute myocardial infarction: a randomised trial. Lancet..

[13] Pickard JM, Davidson SM, Hausenloy DJ, Yellon DM (2016). Co-dependence of the neural and humoral pathways in the mechanism of remote ischemic conditioning. Basic Res Cardiol.

[14] Donato M, Buchholz B, Rodriguez M (2013). Role of the parasympathetic nervous system in cardioprotection by remote hindlimb ischaemic preconditioning. Exp Physiol.

[15] Gourine A, Gourine AV (2014). Neural mechanisms of cardioprotection. Physiology (Bethesda).

[16] Redington KL, Disenhouse T, Li J (2013). Electroacupuncture reduces myocardial infarct size and improves post-ischemic recovery by invoking release of humoral, dialyzable, cardioprotective factors. J Physiol Sci.

[17] Ni X, Xie Y, Wang Q (2012). Cardioprotective effect of transcutaneous electric acupoint stimulation in the pediatric cardiac patients: a randomized controlled clinical trial. Paediatr Anaesth.

[18] Jensen RV, Stottrup NB, Kristiansen SB, Botker HE (2012). Release of a humoral circulating cardioprotective factor by remote ischemic preconditioning is dependent on preserved neural pathways in diabetic patients. Basic Res Cardiol.

[19] Basalay MV, Davidson SM, Gourine AV, Yellon DM (2018). Neural mechanisms in remote ischaemic conditioning in the heart and brain: mechanistic and translational aspects. Basic Res Cardiol.

[20] Sbruzzi G, Silveira SA, Silva DV, Coronel CC, Plentz RD (2012). Transcutaneous electrical nerve stimulation after thoracic surgery: systematic review and meta-analysis of 11 randomized trials. Rev Bras Cir Cardiovasc.

[21] Wang Z, Yu L, Wang S (2014). Chronic intermittent low-level transcutaneous electrical stimulation of auricular branch of vagus nerve improves left ventricular remodeling in conscious dogs with healed myocardial infarction. Circ Heart Fail.

[22] Mo Y, Chen S, Yang L (2017). The effect of transcutaneous electrical Acupoint stimulation on inflammatory response in patients undergoing limb ischemia-reperfusion. Mediat Inflamm.

[23] Johnson M (2007). Transcutaneous electrical nerve stimulation: mechanisms, Clinical Application and Evidence. Rev Pain.

[24] Rossello X, Yellon DM (2016). Cardioprotection: the disconnect between bench and bedside. Circulation..

[25] Rossaint J (2018). Propofol anesthesia and remote ischemic preconditioning: an unfortunate relationship. Anesth Analg.

[26] Behmenburg F, van Caster P, Bunte S (2018). Impact of anesthetic regimen on remote ischemic preconditioning in the rat heart in vivo. Anesth Analg.

[27] Cho YJ, Lee E-H, Lee K (2017). Long-term clinical outcomes of remote ischemic preconditioning and Postconditioning outcome (RISPO) trial in patients undergoing cardiac surgery. Int J Cardiol.

[28] Yang L, Yang J, Wang Q (2010). Cardioprotective effects of electroacupuncture pretreatment on patients undergoing heart valve replacement surgery: a randomized controlled trial. Ann Thorac Surg.

[29] Wang Z, Yu L, Chen M, Wang S, Jiang H (2014). Transcutaneous electrical stimulation of auricular branch of vagus nerve: a noninvasive therapeutic approach for post-ischemic heart failure. Int J Cardiol.

[30] Wang K, Ju Z, Chen C (2020). Cardioprotective effect of electroacupuncture in cardiopulmonary bypass through apelin/APJ signaling. Life Sci.

[31] Tsou MT, Huang CH, Chiu JH (2004). Electroacupuncture on PC6 (Neiguan) attenuates ischemia/reperfusion injury in rat hearts. Am J Chin Med.

[32] Zeng Q, He H, Wang XB, Zhou YQ (2018). Electroacupuncture preconditioning improves myocardial infarction injury via enhancing AMPK-dependent autophagy in rats. Biomed Res Int.

[33] Dong JH, Liu YX, Zhao J, Ma HJ, Guo SM, He RR (2004). High-frequency electrical stimulation of femoral nerve reduces infarct size following myocardial ischemia-reperfusion in rats. Sheng Li Xue Bao.

[34] Brown L, Tabasam G, Bjordal JM, Johnson MI (2007). An investigation into the effect of electrode placement of transcutaneous electrical nerve stimulation (TENS) on experimentally induced ischemic pain in healthy human participants. Clin J Pain.

[35] Schulman D, Latchman DS, Yellon DM (2001). Effect of aging on the ability of preconditioning to protect rat hearts from ischemia-reperfusion injury. Am J Physiol Heart Circ Physiol.

[36] Gao J, Fu W, Jin Z, Yu X (2006). A preliminary study on the cardioprotection of acupuncture pretreatment in rats with ischemia and reperfusion: involvement of cardiac beta-adrenoceptors. J Physiol Sci.

