# Supplementary material for: Effects of transcutaneous electrical nerve stimulation on myocardial protection in patients undergoing aortic valve replacement: a randomized clinical trial
Source: BMC Anesthesiol. 2022 Mar 9;22:68. doi: 10.1186/s12871-022-01611-x (PMC8905743; doi:10.1186/s12871-022-01611-x)
Supplement: Supplementary file 1 — Additional file 1: Supplementary Table 1. Comorbidities, preoperative medications and aortic valve characteristics of the included patients. [file 12871_2022_1611_MOESM1_ESM.docx]

**Supplementary Table 1.** Comorbidities, preoperative medications and aortic valve characteristics of the included patients

| **Characteristics** | **PRE**  **(*n* = 5)** | **s-PRE**  **(*n* = 5)** | **SEVO**  **(*n* = 5)** | **s-SEVO**  **(*n* = 5)** | **PPF**  **(*n* = 5)** | **s-PPF**  **(*n* = 5)** | ***p*-value** |
| --- | --- | --- | --- | --- | --- | --- | --- |
| **Comorbidities** |  |  |  |  |  |  |  |
| Hypertension | 1 (20%) | 4 (80%) | 1 (20%) | 2 (40%) | 1 (20%) | 1 (20%) | 0.375 |
| Diabetes mellitus | 0 (0%) | 2 (40%) | 1 (20%) | 0 (0%) | 0 (0%) | 1 (20%) | 0.658 |
| Coronary artery disease^a^ | 2 (40%) | 1 (20%) | 0 (0%) | 1 (20%) | 1 (20%) | 2 (40%) | 0.674 |
| Previous PCI | 0 (0%) | 0 (0%) | 0 (0%) | 0 (0%) | 0 (0%) | 0 (0%) | N/A |
| Atrial fibrillation | 1 (20%) | 4 (80%) | 0 (0%) | 1 (20%) | 0 (0%) | 0 (0%) | 0.018 |
| Previous stroke | 0 (0%) | 1 (20%) | 1 (20%) | 0 (0%) | 0 (0%) | 1 (20%) | >0.999 |
| ESRD | 0 (0%) | 1 (20%) | 0 (0%) | 0 (0%) | 0 (0%) | 0 (0%) | >0.999 |
| **Preoperative medication** |  |  |  |  |  |  |  |
| Aspirin | 0 (0%) | 0 (0%) | 2 (40%) | 0 (0%) | 2 (40%) | 0 (0%) | 0.111 |
| Clopidogrel | 0 (0%) | 0 (0%) | 1 (20%) | 2 (40%) | 1 (20%) | 0 (0%) | 0.658 |
| ACE inhibitor | 0 (0%) | 0 (0%) | 0 (0%) | 0 (0%) | 0 (0%) | 0 (0%) | N/A |
| ARB | 3 (60%) | 1 (20%) | 1 (20%) | 2 (40%) | 2 (40%) | 2 (40%) | 0.945 |
| CCB | 1 (20%) | 2 (40%) | 0 (0%) | 2 (40%) | 0 (0%) | 0 (0%) | 0.342 |
| BB | 2 (40%) | 2 (40%) | 0 (0%) | 3 (60%) | 1 (20%) | 0 (0%) | 0.270 |
| Diuretics | 1 (20%) | 2 (40%) | 1 (20%) | 3 (60%) | 3 (60%) | 3 (60%) | 0.699 |
| Nitroglycerin | 0 (0%) | 0 (0%) | 0 (0%) | 0 (0%) | 0 (0%) | 0 (0%) | N/A |
| Digoxin | 0 (0%) | 1 (20%) | 0 (0%) | 1 (20%) | 0 (0%) | 0 (0%) | >0.999 |
| OHA | 0 (0%) | 2 (40%) | 1 (20%) | 0 (0%) | 0 (0%) | 0 (0%) | 0.384 |
| Insulin | 0 (0%) | 0 (0%) | 0 (0%) | 0 (0%) | 0 (0%) | 1 (20%) | >0.999 |
| Statin | 1 (20%) | 3 (60%) | 3 (60%) | 4 (80%) | 3 (60%) | 3 (60%) | 0.699 |
| **Preoperative Aortic valve characteristics** |  |  |  |  |  |  |  |
| AV stenosis | 4 (80%) | 4 (80%) | 3 (60%) | 3 (60%) | 4 (80%) | 2 (40%) | 0.875 |
| AV regurgitation | 0 (0%) | 0 (0%) | 1 (20%) | 0 (0%) | 0 (0%) | 2 (40%) | 0.384 |
| AV stenosis combined with regurgitation | 1 (20%) | 1 (20%) | 1 (20%) | 2 (40%) | 1 (20%) | 1 (20%) | >0.999 |
| Etiology of AV disease |  |  |  |  |  |  | 0.947 |
| Degenerative | 2 (40%) | 3 (60%) | 2 (40%) | 2 (40%) | 1 (20%) | 2 (40%) |  |
| Rheumatic | 1 (20%) | 1 (20%) | 2 (40%) | 1 (20%) | 1 (20%) | 2 (40%) |  |
| Bicuspid | 2 (40%) | 1 (20%) | 1 (20%) | 2 (40%) | 3 (40%) | 1 (20%) |  |
| AV peak velocity (m/s) | 4.5 ± 1.1 | 4.2 ± 0.2 | 4.2 ± 0.6 | 4.9 ± 0.9 | 4.8 ± 0.8 | 3.6 ± 1.2 | 0.329 |
| AV mean pressure gradient (mmHg) | 48.9 ± 19.4 | 40.5 ± 4.0 | 41.9 ± 11.3 | 59.7 ± 20.7 | 56.2 ± 17.7 | 35.8 ± 22.3 | 0.249 |
| AV area (cm^2^) | 1.0 ± 0.5 | 0.9 ± 0.2 | 0.8 ± 0.0 | 0.8 ± 0.3 | 0.7 ± 0.2 | 1.2 ± 0.8 | 0.526 |

Data are presented as mean ± SD, median (interquartile range [range]), or number (%).

TENS, transcutaneous electrical nerve stimulation; SEVO, sevoflurane; PPF, propofol; PCI, percutaneous coronary intervention; ESRD, end-stage renal disease; ACE, angiotensin converting enzyme; ARB, angiotensin receptor blocker; CCB, calcium channel blocker; BB, beta-blocker; OHA, oral hypoglycemic agent; AV, aortic valve; AVR, aortic valve replacement; CABG, coronary artery bypass graft.

^a^ Coronary artery disease was determined by preoperative coronary angiography or computed tomography coronary angiography.
